# Freeman–Sheldon Syndrome: First Molecularly Confirmed Case from Sub-Saharan Africa

**DOI:** 10.1155/2017/9327169

**Published:** 2017-05-11

**Authors:** A. M. Ali, R. M. Mbwasi, G. Kinabo, E.-J. Kamsteeg, B. C. Hamel, M. C. J. Dekker

**Affiliations:** ^1^Department of Paediatrics and Child Health, Kilimanjaro Christian Medical Centre, P.O. Box 3010, Moshi, Tanzania; ^2^Department of Human Genetics, Radboud University Medical Center, Geert Grooteplein Zuid 10, 6525 GA Nijmegen, Netherlands; ^3^Department of Neurology, Radboud University Medical Center, Geert Grooteplein Zuid 10, 6525 GA Nijmegen, Netherlands

## Abstract

We report a case of a male baby who has characteristic signs of Freeman–Sheldon syndrome, a rare but recognizable, severe autosomal dominant form of distal arthrogryposis. Diagnosis was based on the distinctive clinical characteristics of the syndrome and confirmed by genetic analysis that showed a de novo missense mutation c.2015G>A (p.Arg672His) of the* MYH3* gene. We highlight the different features present in our patient and describe the etiology of the Freeman–Sheldon phenotype and how its clinical complications can be dealt with. To the best of our knowledge, this is the first molecularly confirmed case of Freeman–Sheldon syndrome in sub-Saharan Africa.

## 1. Introduction

First reported in 1938 by Freeman and Sheldon who initially called it craniocarpotarsal dystrophy [1], Freeman–Sheldon syndrome (FSS; OMIM #193700), also known as craniocarpal-tarsal dysplasia, windmill vane hand syndrome, distal arthrogryposis type 2A, and whistling-face syndrome, is a rare form of multiple congenital contracture syndrome. It is inherited in an autosomal dominant pattern [2] with most cases occurring sporadically with no family history of the disease. It is characterised by facial and distal limb contractures, the most common being microstomia with pouting lips, camptodactyly with ulnar deviation of the fingers and talipes equinovarus. Facial characteristics comprise micrognathia, microglossia, high arched palate, vertical skin folds in the jaw, H-shaped chin dimple, and a characteristic “mask-like” facies. Other features are dental crowding, strabismus, and hearing loss. Kyphoscoliosis may present later in life. Speech and motor development are delayed; however, cognitive development and life expectancy are usually normal. To date about 100 individuals bearing the characteristic clinical phenotype have been reported [3]. The genetic basis was unravelled in 2006 with missense mutations in the motor domain of the embryonic myosin heavy chain* MYH3* gene, which encodes embryonic myosin, one of the proteins of the contractile complex of skeletal muscle cells [4].

In the African continent, FSS has already been reported in Tanzania [5], South Africa [6], and Egypt [7]. To the best of our knowledge, we hereby describe the first molecularly confirmed FSS patient from sub-Saharan Africa, in whom* MYH3* mutation analysis revealed a recurrent pathogenic missense mutation.

## 2. Case Report

A newborn male was shortly after birth referred to our hospital. He was brought in by the mother with a main complaint of abnormal facial, genital, upper, and lower limb features since birth. He was born at term with a gestational age of 39 weeks by spontaneous vaginal delivery, a birth weight of 3.1 kg, and an APGAR score of 5 and 7 in the 1st and 5th minute, respectively. He is the 4th child born to healthy and nonconsanguineous parents. All other children are alive and developing well and none have congenital abnormalities. Currently, the mother is 36 years old and the father is 45 years old. The mother consumed alcohol about once or twice a month during pregnancy, never excessive. Pedigree analysis showed that none of the other family members had the same anomalies.

**Figure 1:**
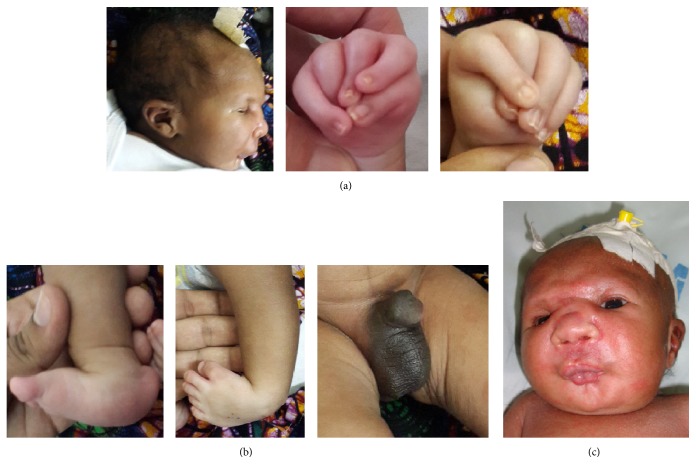
(a) from left to right shows micrognathia and low-set crumpled ears, camptodactyly of the right hand, and camptodactyly and “windmill vane position” of the left hand. (b) from left to right shows congenital vertical talus of the right foot (rocker bottom foot), Talipes equinovarus of the left foot (club foot) and chordee. (c) shows the “mask-like” face with a wide and flat nasal bridge, ocular hypertelorism, microstomia, and puckered lips (“whistling face”).

On inspection, an active newborn was seen with a normal level of consciousness and pink in room air. He was hypothermic (33.5°C) and tachypneic (77 bpm) with an appropriate heart rate for the age (147 bpm). As shown in Figure 1, he had multiple craniofacial abnormalities including micrognathia and low-set ears, a wide and flat nasal bridge, ocular hypertelorism, squinted eyes, microstomia, and puckered lips that looked like as if he was whistling. Multiple contractures including camptodactyly of both hands with fingers in the “windmill vane position,” a right congenital vertical talus, and left talipes equinovarus were noted. On genitourinary examination, chordee (downward curved penis) was seen without hypospadias. Initially, expressed breast milk was given through a nasogastric tube. Additional investigations such as brain and cardiac ultrasound were ordered; however they could not be performed due to financial constraints of the mother. Later, the mother was taught how to feed the baby through a container using expressed breast milk and counselled to initiate correction of the talipes equinovarus using the Ponseti method. The child was discharged on day 10 and followed up at the Departments of Physiotherapy and Paediatrics.

Based on the history and physical examination, the clinical diagnosis was FSS. Venous blood was sampled and sent to the Genome Diagnostics Nijmegen of Radboud University Medical Center, Nijmegen, Netherlands, for confirmation of the clinical diagnosis.

DNA sequence analysis of the* MYH3* gene revealed a heterozygous missense mutation c.2015G>A (p.Arg672His), which was absent in both parents. This mutation is pathogenic because it is de novo, has been published already [4], and was not observed in the exomes of more than 60000 presumably healthy individuals according to the ExAc browser (http://exac.broadinstitute.org).

## 3. Discussion

Distal arthrogryposis (DA) is an inherited primary malformation involving multiple congenital contractures of the upper and lower limbs, for example, camptodactyly, absent/hypoplastic flexion creases, overriding fingers, talipes equinovarus, calcaneovalgus deformities, and vertical talus. Nine groups have been classified by Bamshad et al., each one numerically labelled in order of similarity to DA1 which was taken as the model disorder; that is, DA2 is more similar to DA1 than is DA9 [8]. In addition to the congenital contractures of the upper and lower limbs each DA has distinguishing characteristic features unique to that form of DA.

FSS (DA2A) is the most severe form of the distal arthrogryposes while Sheldon-Hall syndrome (SHS, DA2B) is the most common. There is a significant phenotypical overlap between SHS and FSS often causing misdiagnoses between the two, particularly in children. While both FSS and SHS have orofacial manifestations, SHS is lacking a whistling face and H-shaped dimpling of the chin [4, 9]. In contrast to FSS, there is genetic heterogeneity in SHS in which, apart from* MYH3* mutations, there are also mutations observed in* TNNI2*,* TNNT3*, and* TPM2* [9, 10]. In 2006 strict clinical criteria for classical FSS were published by Stevenson et al. which included the presence of two or more of the clinical manifestations of DA described above plus the presence of a small pinched mouth, prominent nasolabial folds and H-shaped dimpling of the chin [11]. Since all these criteria were apparent in our patient, it was not very difficult to reach a provisional diagnosis. However, there is a broad spectrum of variability in the clinical presentation of FSS to the extent that only orofacial manifestations of the disease can be present without abnormalities of the limbs [12]; thus genetic analysis is often needed to reinforce the diagnosis.

FSS is caused by a mutation of the embryonic myosin heavy chain* MYH3* gene, which is present on the short arm of chromosome 17. MYH3 protein aids in the production of the skeletal muscle cells which is vital for normal development of muscles before birth. MYH3 expression has been seen to be downregulated in skeletal muscle when nearing the end of gestation in humans [13], suggesting that the contractures are not progressive after birth and will not influence contractile function postnatally. However, a more recent study has shown conflicting results [14]. Genetic analysis of our patient revealed the most common mutation found in FSS which is the heterozygous missense mutation c.2015G>A (p.Arg672His), which accounts for around 45% of* MYH3* mutations in FSS.

A genotype-phenotype correlation study revealed that by using a severity score system (ranging from 0 to 37) that takes into account the physical findings of the upper and lower limbs and face, as well as the natural history of DA [15], the p.Thr178Ile mutation gives the most severe phenotype and the p.Arg672Cys mutation the least severe phenotype, while the p.Arg672His mutation shows intermediate severity. Together these 3 mutations account for more than 90% of the* MYH3* mutations in FSS [16]. Beck et al. found that the severity scores of patients with the p.Arg672His mutation ranged from 4 to 15 with a mean of 9.2. Most of the clinical variability appeared to be in the lower limb features [16].

Mutations can either occur sporadically in every first case of FSS in a family—as in our case—or it can be passed on from one generation to the next as an autosomal dominant trait [2] with a 50 percent chance to pass it on to the offspring. Occurrences of FSS in sibships with normal parents (but without molecular confirmation) suggests an autosomal recessive pattern [17–19] and, with 2 affected brothers, X-linked recessive inheritance has also been reported [20]. Recently, mosaicism in a phenotypically normal parent of a girl with FSS has been observed [21]. As already suggested by Stevenson et al. [11], these cases could well have been due to parental mosaicism.* MYH3* mutations are not exclusive to FSS alone. These have also been found in SHS (DA2B) [4] and multiple pterygium syndrome (DA8) [22] and quite recently in autosomal dominant spondylocarpotarsal synostosis syndrome [23].

Obviously, management of such a complex disorder as FSS requires a multidisciplinary approach, starting from birth. Because of the characteristic orofacial manifestations like microstomia (pouting lips), microglossia, and micrognathia, many newborns with FSS experience feeding difficulties due to the inability to form a tight seal around the nipple. Exclusive breast feeding rarely works; hence alternatives like using specialized bottle-top nipples, orogastric or nasogastric tubes, gastrostomy tubes, or expressing breast milk and feeding the baby using either a small container or spoon have been used for the first months of life or even longer. Regardless of the method used, commissurotomy has shown improvement in the ability to feed [11]. Bilateral commissurotomy followed by the use of a patient customized dynamic oral commissure retractor has been effective in dealing with microstomia and preventing recurrence due to scar contracture that occurs months following surgical intervention [24].

Distal limb contractures should be dealt with by early physiotherapy where manipulative techniques such as the Ponseti method can be used for talipes equinovarus. In case of refractory early intervention, surgical correction can also be employed. Unlike talipes equinovarus, vertical talus rarely responds to stretching and casting alone; however, a combination of early serial manipulations and casting followed by limited surgical correction by tenotomy and k-wires has shown to be successful [25].

Patients with FSS require numerous hospital admissions and surgeries throughout life averaging 17 and 10.3 per individual, respectively [11]. The aim of these surgeries is to try to correct the phenotypical manifestations. Some of these surgeries are most effectively performed at particular age groups, for example, commissurotomy should be done earlier to overcome feeding difficulties and speech delays whereas corrective surgical procedures for limbs can be done later, once conservative management has failed. Therefore, counselling parents/guardians regarding all these management issues is of paramount importance.

Numerous complications can occur during surgery on patients with FSS. The most common is difficult intubation [26, 27], secondary to the microstomia, high arched palate, and micrognathia which does not improve with neuromuscular blockade or general anaesthesia [28]. The uses of a laryngeal mask airway [29] for short procedures and fibre-optic bronchoscopy [30] have both been successful in the difficult airway management of these patients. Another complication observed multiple times in patients with FSS is malignant hyperthermia [26, 31] with a frequency of 16% as reported by Stevenson et al. [11]. It normally develops with use of volatile anaesthetics, for example, in halothane induction with or without the use of muscle relaxants like succinylcholine. It is a condition in which muscles enter a hypermetabolic state resulting in elevated temperature as a consequence of heat production, increased heart rate, acidotic breathing, muscle rigidity, and rhabdomyolysis which can lead to acute kidney injury and eventually to multiple organ dysfunction syndrome and death, if not treated aggressively. Hence, when surgery is considered, volatile anaesthetics and depolarizing muscle relaxants should be avoided and it should be done at facilities that are geared to deal with these complications, should they arise.

In conclusion, FSS is a rare but recognizable, generally severe, autosomal dominant type of DA, due to mutations in the* MYH3* gene. We described the first FSS patient from sub-Saharan Africa, in whom a* MYH3* mutation was found. Patients with FSS should be evaluated and managed from birth by healthcare professionals from multiple fields of medicine including paediatricians, dentists, anaesthesiologists, orthopaedic and craniofacial surgeons, physiotherapists, and occupational therapists in order to gauge the severity of their phenotypical presentation and create an appropriate personalized management plan.
